# Ductal Hyperkeratinization and Acinar Renewal Abnormality: New Concepts on Pathogenesis of Meibomian Gland Dysfunction

**DOI:** 10.3390/cimb45030122

**Published:** 2023-02-27

**Authors:** Ya-Li Du, Xi Peng, Yang Liu, Jia-Song Wang, You-Fan Ye, Kang-Kang Xu, Jing-Yu Qu, Hua Chen, Hua-Tao Xie, Ming-Chang Zhang

**Affiliations:** 1 Department of Ophthalmology, Union Hospital, Tongji Medical College, Huazhong University of Science and Technology, Wuhan 430022, China; 2 Department of Ophthalmology, Zhongnan Hospital of Wuhan University, Wuhan 430071, China

**Keywords:** meibomian gland dysfunction, meibocyte, hyperkeratinization, stem cell, pathogenesis

## Abstract

Meibomian gland dysfunction (MGD) is a functional and morphological disorder of the meibomian glands which results in qualitative or quantitative alteration in meibum secretion and is the major cause of evaporative dry eye (EDE). EDE is often characterized by tear film instability, increased evaporation, hyperosmolarity, inflammation, and ocular surface disorder. The precise pathogenesis of MGD remains elusive. It has been widely considered that MGD develops as a result of ductal epithelial hyperkeratinization, which obstructs the meibomian orifice, halts meibum secretion, and causes secondary acinar atrophy and gland dropout. Abnormal self-renewal and differentiation of the acinar cells also play a significant role in MGD. This review summarizes the latest research findings regarding the possible pathogenesis of MGD and provides further treatment strategies for MGD-EDE patients.

## 1. Introduction

Meibomian glands (MGs) were first described by Heinrich Meibom in 1666 [1]. They are modified, holocrine glands in the upper and lower eyelids and are the largest sebaceous glands in the human body [2]. Twenty to thirty MGs are located in the lower lid, and thirty to forty are located in the upper lid [3]. Meibum is secreted onto the ocular surface by the MGs, forming the lipid layer of the tear film, which plays a role in preventing the aqueous tear from evaporating, isolating pathogenic microorganisms, maintaining the stability of the tear film, and assisting in the tight closure of the lid margin during night sleep [4].

Meibomian gland dysfunction (MGD) is a chronic, diffuse disorder of the MGs, typically presented with terminal duct blockage and/or qualitatively/quantitatively altered gland production [5]. The prevalence of MGD is higher in Asian populations, with 40–70%, with a lower incidence in Caucasian populations, ranging from 3.5% to 20% [6]. It is the leading cause of evaporative dry eye (EDE) [7]. MGD-EDE can be identified by the presence of an unstable tear film, which causes the accelerated evaporation of aqueous tears, the increased osmolarity of the tear film, and posterior blepharitis [8]. Over the past few years, two main pathogenic mechanisms, ‘ductal centric’ and ‘meibocyte centric’ hypotheses, have been proposed [9,10,11]. The former holds the view that MGD involves the hyperkeratinization of the ductal epithelium and causes the obstruction of the ducts, orifice closure, glandular stasis, cystic dilation, and subsequent atrophy of the holocrine, excretory acini [11,12]. Jester et al. have proposed that the self-renewal of acinar cells plays a significant role in MGD. The abnormal proliferation and differentiation of meibocytes are the main causes of MGD rather than the hyperkeratinization of the ductal system [9,10,13]. Recent studies from our laboratory suggested that the co-effects of the hyperkeratinization of the ducts and the abnormal self-renewal of the acinar cells lead to MGD [14,15,16]. In addition, bacteria have a critical role in the occurrence of MGD [17,18,19].

In this review, we will provide our current insights about the pathogenesis of MGD and therapeutic methods based on these new concepts.

## 2. Physiology of MG

**Figure 1:**
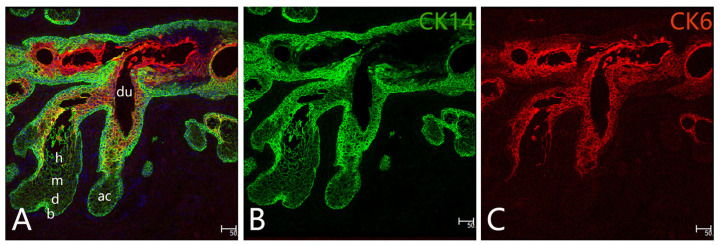
Histology and anatomy of human MG. Structure of the acinar (ac) and ductal (du) system of a normal MG (**A**). The meibocytes go through four maturation stages: basal (b), differentiating (d), mature (m), hypermature (h). Identification of CK14 and CK6 in the MGs (**B**,**C**): CK14 is observed in both ductal and acinar epithelial cells (**B**), CK6 is only expressed in ductal epithelial cells (**C**).

The MG is a compound duct–acini structure, which consists of linear arrays of sebaceous glands in the tarsal plates of the eyelids [20]. The secretory acini are connected to the central duct by a small duct, which then extends to the tarsal plate and eventually opens into the eyelid [20]. Histologically, meibocytes undergo four different stages of maturation: basal, differentiated, mature, and hypermature [21] (Figure 1A). Basal meibocytes are undifferentiated cells which have the function of proliferation. During the process of differentiation, meibocytes begin to accumulate lipids and undergo apoptosis. Subsequently, the meibocytes experience the shrinkage, compaction, and disintegration of the nucleus (pyknosis). Eventually, the lipids and other cellular components are released through a connecting duct into the central duct [11,22]. Through the gland orifice at the mucocutaneous junction of the lid margin, the lipids and cellular components are then excreted as meibum onto the ocular surface [23]. The ductal system is consisted of a four-layer squamous epithelium [24]. Using electron microscopy, researchers have observed a dramatic transformation from the peripheral layer of basal meibocytes into ductal epithelium in animal models; however, the basal meibocytes transform gradually into multilayered ductal epithelial cells in humans [11]. The MG terminal opens into the free lid margin, lined with the cornified epithelium, and the meibum is secreted from the gland orifice [25].

### 2.1. MG Stem Cells

MGs share a similar biology and cell dynamics with sebaceous glands. In the MGs, cell replacement is continuous due to holocrine secretion, which relies on the MG stem cell dynamic activity [26]. There has been a growing controversy regarding the location and description of stem cells in MGs. Some researchers hypothesized that the MG stem cells lie in the central duct [27,28], whereas several studies have supported the idea that the stem cells are located at the junction between the ducts and the acini [10,29]. Maskin et al. hypothesized that stem cells may be found surrounding each acinus [30]. Geraint et al. illustrated that it is possible for label-retaining cells to govern the turnover of MGs, and these cells could be identified as two distinct unipotent progenitors that generate ducts and acini independently [31]. According to the study by Edem et al., KROX20 labels a stem/progenitor cell population that differentiates to produce MGs [32]. Chen et al. found the differences in the components of the extracellular matrix (ECM) surrounding the MG acini, ducts, and the junctions between them [26]. To further investigate MG stem cells, a better understanding of the localization of different ECM components within the MGs is required. Based on our previous study, Lrig1 was firstly identified as a marker of self-renewing stem/progenitor cells of the MGs, and Lrig1-positive cells are located at the periphery of the MG acini [14]. In the future, more research is required to further study the location, function, renewal, and culture lines of stem cells in MGs.

### 2.2. Differentiation and Proliferation of Meibocytes

MGs consist of three main kinds of epithelial cells, including progenitor, differentiated, and ductal epithelial cells [11]. The acinar system is composed of four stages of meibocyte clusters and links to a central excretory duct via a smaller duct at the junction (Figure 1A) [11]. The meibocytes which are located around the acini will migrate toward its center and then move to the entrance of the duct [11]. During the maturation and differentiation of meibocytes, the cell nuclei disintegrate, and the lipid droplets become larger [33,34]. The meibocytes are constantly renewed by the surrounding basal cells [14]. Ki67 labeling revealed that many basal acinar cells proliferated, although a decreased rate of proliferation was seen in older animals [35,36]. Meibocytes exhibit epithelial cytokeratin (CK) 5, 8, 14, B-lymphocyte-induced maturation protein-1 (BLIMP1), and CD147 [21]. Our previous study [14] indicated that CK14 was expressed in both ductal and acinar epithelial cells (Figure 1B), while CK6 was only expressed in MG ductal cells (Figure 1C).

Based on our recent finding, DNase2 is a differentiation marker expressed in the central differentiated epithelial cells of the MGs but not in the peripheral undifferentiated cells [14]. It has been demonstrated that the self-renewal of acinar cells might be regulated by the Hedgehog pathway [16], which is a family of secreted proteins. It plays a role in regulating the genes of transcription involved in proliferation and differentiation [37]. Inhibition of the Hedgehog pathway leads to a significant decrease in the proliferation of acinar cells, while differentiation and lipid synthesis are increased [16]. The peroxisome-proliferator-activated receptor γ (PPAR γ) nuclear hormone receptor acts as a transcription factor upon ligand activation, which regulates the self-renewal of meibocytes [34]. The expression of PPAR γ varies with age [31]. Differentiation and proliferation are mediated by the downregulation of genes involved in replication and the G1/S transition, as well as by the upregulation of growth signaling pathways and autophagy induction [11]. Over the past few years, several signaling pathways have been proposed [16,38,39,40,41,42]. These have been summarized in Table 1.

### 2.3. Regeneration of MGs

As acinar cells degenerate, new cells are continuously produced, causing a turnover and differentiation of cells within the acinus. The consensus was previously made that MG atrophy was permanent [43]. Yang et al. indicated that MG homeostasis depends on the fibroblast growth factor receptor 2 (FGFR2) gene, which may be an effective method for the regeneration of atrophic acini in MGD patients [33]. Recovery of MGs is proportional to the degree of ductal atrophy, which suggests that ductal epithelia could provide the progenitor cells necessary for acinar regeneration [33]. By changing culture conditions, differentiated immortalized human meibomian gland epithelial cells (IHMGEC) can dedifferentiate into proliferative cells, and with the right stimulus, these cells can redifferentiation again [14,44]. Epithelial cells can be dedifferentiated into an undifferentiated proliferating state, with reversible switching between the two states [14]. The potential of regenerating MGs is promising, although further research is necessary to reinforce these early findings.

## 3. Meibography and Function of MGs

**Figure 2:**
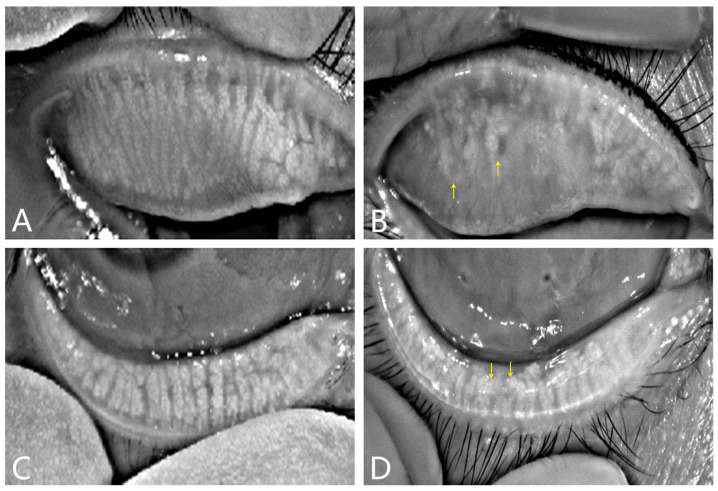
Representative images using noninvasive infrared meibography. Normal MGs of a young man show columnar and relatively straight glands, with those in the upper eyelid (**A**) being slenderer and more elongated than those in the lower eyelid (**C**). MGD individual with severe MG atrophy of the upper eyelid ((**B**), yellow arrow) and mild MG atrophy in the lower eyelid ((**D**), yellow arrow).

The diagnosis of MGD relies on morphological and functional assessments. Currently, a variety of morphological methods have become available, including meibography by transillumination of an everted lid at the slit lamp, in vivo microscopy (IVCM), and noncontact infrared photography. The slit lamp examination is an economical and effective way to observe the tear film break time (BUT), the secretion of meibum, the closure of the MG orifice, and the hyperkeratinization of the lid margin [45]. IVCM could detect the alternation of MGs at the cellular level and provide a pathophysiological system, but it is contact-sensitive and may cause discomfort to the patients [46]. Meibography is important for capturing the images of MGs in real time. Traditionally, the devices illuminated the tarsal plate from the skin side for seeing the structure of the MGs [47,48]. With the advancement of technology, noncontact infrared photography has been used to assess MG morphology, including BG-4M/DC-4 (Topcon, Tokyo, Japan), the Meibom Pen (Japan Focus Corporation, Tokyo, Japan), the Keratograph 4 (K4) (Oculus, Wetzlar, Germany), the Keratograph 5M (K5M) (Oculus, Wetzlar, Germany), the Meiboviewer 2.0 (Visual Optics, Chuncheon, Korea), and the Cobra HD fundus camera (Scandicci, Firenze, Italy) [49,50,51,52]. In normal subjects, relatively straight glands can be observed, with those in the upper eyelid (Figure 2A) being slenderer and more elongated than those in the lower eyelid (Figure 2C). Glandular atrophy can be seen in patients with MGD (Figure 2B and D, yellow arrows). Various studies have investigated the correlation between meibography score and parameters related to dry eye, such as the lipid layer thickness of the tear film, the noninvasive breakup time (BUT) of the tear film, the ocular surface disease index (OSDI), and glandular atrophy [53,54]. The K4 and K5M show similar results, indicating that the meibography score is related to the degree of gland dropout instead of clinical signs [54]. Different grading systems of morphology have been proposed for the evaluation of the severity of MGD, but morphological evaluation is still insufficient to diagnose MGD [55]. Recent studies mainly focus on changes in the morphology of MG with age [49], sex [56], allergic diseases [57], drug use, and contact lens wear [58].

## 4. Pathological Mechanism of MGD

MGD results from abnormal MG function and/or morphology, as commonly shown by alternations in the quality and quantity of the meibum [11]. Both primary and secondary causes can lead to MGD [59]. It can be associated with different systemic diseases such as diabetes mellitus, hypercholesterolemia, atopy, and atopic dermatitis [60]. MGD usually results in changes in the tear film, irritation, inflammation, and ocular surface disease [11]. It is commonly classified as low delivery or high delivery according to the amount of meibum secretion [2]. The low delivery MGD is usually due to the primary hyposecretion of acinar cells or obstruction of the MG ductal system [2]. Conversely, high-delivery states are defined as the excessive production of meibum in response to eyelid pressure at the lid margin, resulting in the hyperkeratosis of the duct epithelium, the deposition of keratinous debris, and the increased viscosity of the lid fluid [11]. It is always associated with seborrheic dermatitis or ocular rosacea [11].

There are two main hypotheses for pathological mechanisms, namely the ‘ductal centric’ and ‘meibocyte centric’ [9,10,11,61,62]. Traditionally, the underlying pathophysiology is epithelial hyperkeratinization, which causes the obstruction of the ductal system, the stasis of meibum, cystic dilation, and eventually disuse acinar atrophy and gland dropout [11,61]. Recent research has expanded on this paradigm, describing acinar atrophy resulting from abnormalities in meibocyte differentiation and self-renewal as a contributing mechanism in MGD [9,10,62]. We have summarized the pathological mechanisms of MGD in Table 2.

### 4.1. Ductal-Centric Theory

**Figure 3:**
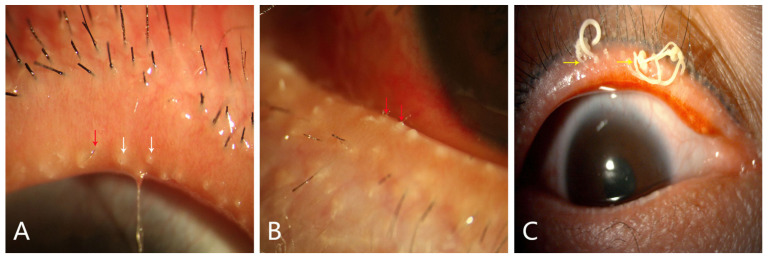
Eyelid of a 29-year-old female with MGD-EDE, showing keratinized marginal conjunctiva, inspissation of duct orifices ((**A**), white arrows) with hair growth from MGs ((**A**,**B**), red arrows). A 35-year-old female with MGD shows expressible highly viscous meibum ((**C**), yellow arrow).

Korb and Henriquez first described obstructive MGD as a result of hyperkeratinization in patients [63]. A noteworthy discovery was the overexpression of keratinization- related genes, including genes coding for small proline-rich proteins (SPRRs) and calcium binding proteins S100 (S100 A7, A8, and A9) [61]. Increased expression of genes involved in keratinization leads to the hyperkeratinization of the ductal system, which leads to altered meibum flow and plug formation [64]. According to the researchers, the gland orifice, central ducts, and meibum themselves exhibit abnormal keratinization in patients with MGD [64]. Obstructive MGD revealed a keratinized marginal conjunctiva, blocked orifice and keratotic clusters that included desquamated epithelium and thickened meibum [64]. From clinical observations, inspissation of the duct orifices (Figure 3A, White Arrows); hair growth from the MGs (Figure 3A,B, Red Arrows); expressible, highly viscous meibum (Figure 3C, Yellow Arrow); and the obstruction of the excretory duct by increased keratinization are observed in MGD-EDE patients. As a consequence of the dilatation and obstruction of the ductal system, acinar degeneration and atrophy were observed, providing evidence of insufficient secretion due to loss of secretory glands [11]. Various cell cultures for MG derived from rabbits, mice, and humans have been developed in recent years [22]. Based on our earlier research, organotypic culture of mouse MG was successfully established, and the process of IL-1β-induced hyperkeratinization of ducts was observed in vitro [65].

### 4.2. Meibocyte-Centric Theory

Continual secretion may cause the atrophic degeneration of the MGs and the gradual loss of lid tissues. With increasing age, the Ki67 labeling of the basal meibocyte layer demonstrated a substantial decrease in mitosis in proliferating cells [11]. This acinar atrophic process results in a decrease in oil production, which eventually leads to the instability of the tear film and symptoms of ocular surface irritation [29]. It is assumed that MGD has a primary, age-dependent acinar form that causes glandular dysfunction over time [66]. PPARγ has been widely studied and confirmed to be involved in the regulation of morphogenesis, function, and meibocyte differentiation [34]. It has been observed that the expression of PPARγ in acinar cells is different between young patients and old people, with obvious cytoplasmic and nuclear localization in younger tissues [10]. Age-related changes in the expression of PPARγ and the self-renewal of meibocytes showed a consistent conclusion between animal models and human MGs [35,36]. Based on the Hwang et al. study, the hyperkeratinization of the ductal system was not detected in an age-related mouse model [10]. The gene analysis of PPARγ showed a significant decrease with aging, while there was no significant difference in the expression of keratinization-related genes [67].

## 5. Endogenous and External Factors for MGD

Age, sex, hormonal disturbance, and environmental factors play a role in the regulation of the process of ductal hyperkeratinization and meibocyte renewal and differentiation. Age-related MGD is associated with decreased meibocyte cell renewal and lipid synthesis via the SRC (a nonreceptor tyrosine kinase protein that is encoded by the SRC gene in humans) pathway [41]. According to a previous study, the alternation of age-related cell signaling within the MG can result in gland atrophy [62]. Androgens are known to regulate sebaceous gland development, differentiation, and lipid production throughout the body [68]. It has been reported that androgens affect the expression of keratinization genes of MGs and regulate pathways involved in lipid dynamics and peroxisome-proliferator-activated receptor (PPAR) signaling [69]. Environmental factors can also contribute to MGD. In animal models, low humidity stress and particulate matter 2.5 (PM 2.5) cause several meibocyte-associated abnormalities, including an increase in basal acinar cell proliferation, an altered meibum protein-to-lipid ratio, irregular meibocyte differentiation, and meibocyte stem cell depletion [10,70]. In addition, bacterial colonization is another important factor that accounts for MGD. Several studies have demonstrated that bacteria such as *S. aureus* [15] and *malassezia* species [71] might alter meibum viscosity and cause stasis in the MG, resulting in inflammation and hyperkeratinization [11]. During the development of MGD, bacterial byproducts such as toxins and lipase play a role in the pathogenesis of MGD. Bacterial lipase may modify the lipid content by affecting the physical properties of the mucous layer and inducing MGD-EDE [72]. MGs can also be altered through external factors such as contact lens wear [73] and some congenital disorders, such as Turner syndrome [29].

## 6. Our Perspectives for MGD

**Figure 4:**
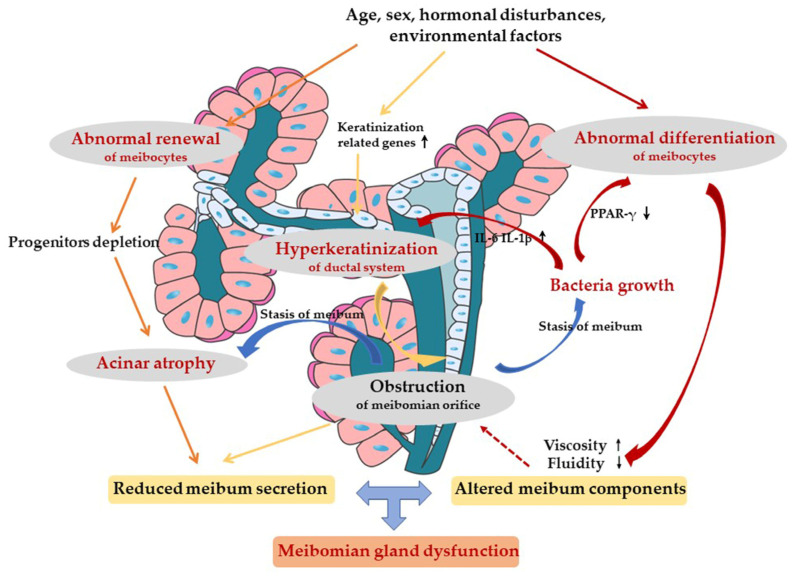
Pathogenesis of MGD occurs as a result of hyperkeratinization of the ductal system and abnormal renewal and differentiation of meibocytes. Both endogenous and external factors, such as age, sex, hormonal disturbances, and environmental factors, have effects. Abnormal renewal of meibocytes leads to progenitor cell depletion and further acinar atrophy. Hyperkeratinization of the epithelium of the excretory duct leads to the obstruction of the MG orifice, the stasis of meibum and secondary acinar cells atrophy. Stasis of meibum can further lead to the growth of bacteria. The bacteria increase the expression of proinflammatory cytokines, including IL-1β and IL-6, which can induce inflammation of the ocular surface. Bacteria also play an essential role in inhibiting the PPARγ pathway to influence the differentiation of meibocytes, leading to altered meibum components. Increased viscosity and decreased fluidity of meibum could in turn reinforce the obstruction. All these changes lead to the qualitative/quantitative changes in gland secretion, resulting in MGD eventually.

Based on previous findings, we propose that both the hyperkeratinization of the ductal system and the abnormal self-renewal of acinar cells contribute to the development of MGD, which can be regulated by intrinsic and extrinsic factors [14,15,16,65,74]. The Hedgehog pathway plays an important role in the proliferation and differentiation of meibocytes [16]. Inhibition of the Hedgehog pathway decreased acinar cell proliferation while increasing differentiation and lipid synthesis [16]. *S. aureus* could lead to the hyperkeratinization of the entire MGs, both in the acinar and ductal systems. It decreased the dynamic activity of meibomian glandular tissue, promoted cell death, inhibited cell proliferation, and destructed the morphology of the acinar and duct. In addition, it downregulated the expression of PPARγ, which is crucial for lipid synthesis and inflammation inhibition [15].Figure 4 presents the pathological mechanism of MGD.

## 7. Prospective Future Research and Therapeutic Strategies

The pathological features of MGD are critical in the determination of optimal treatments. Recently, various therapies have been proposed, including eyelid hygiene, MG warming and massage, anti-inflammatory and antibiotic medications [75,76]. Eyelid hygiene, warming, and massage can reduce the growth of bacteria and increase the melting point of meibum, relieving the increased meibum viscosity [77]. Yeo et al. demonstrated that the application of Lipiflow could reduce the tear evaporation rate from baseline to 3 months in patients with MGD [78]. At present, instruments for lid margin cleaning are constantly being updated and are showing good results on patients [79,80,81]. Anti-inflammatory and antibiotic medications are also useful for the treatment of MGD as they inhibit the bacterial proliferation, preventing the process of inflammation-induced keratinization of MGs; currently, the most widely used medications are tetracycline and azithromycin [82,83]. Numerous studies have demonstrated the efficiency of topical antibiotics [84,85], with the latest evidence indicating that the act is nonpersistent [86]. In the past few years, perfluorohexyloctane has gained popularity and been widely used in developed countries as an alternative therapy for MGD-EDE [87,88,89]. The possible pharmacological mechanism is that it can serve as a surfactant, forming monolayers at the water/air interphase and preventing the evaporation of the tear film [89]. AZR-MD-001 is currently being studied to evaluate the safety, efficacy, and tolerability in patients with MGD. It mainly targets to break down the bonds between abnormal keratin proteins to soften the blockage, slow down the production of keratin and increase the quality and quantity of meibum [90]. However, the exact effects need to be further studied and evaluated. The chronic condition of MGD has led to the rise of many in-office treatments, which can be implemented by ophthalmologists and optometrists in different parts of the world [83,91].

In summary, MGD is a major cause of ocular surface diseases such as EDE. Previous evidence has confirmed that the pathogenesis of MGD is linked to the alternation of meibum lipids and meibum proteins. Nowadays, more and more researchers are paying attention to protein production, deposition, and aggregation. So far, there has not been a consistent and thorough discussion of the pathophysiology of MGD. It remains unknown whether changes in the MG structure result in alterations in the lipid secretions and whether those changes can be reversed or halted. In the pathogenesis of MGD, the localization and function of MG stem cells need further study. Understanding meibocyte renewal and the maintenance of meibocyte stem cells may enable the treatment of MGD in patients with meibocyte atrophy. A better understanding of the pathological mechanisms helps ophthalmologists and optometrists in effective decision making.

## Figures and Tables

**Table 1:** Pathways mediating differentiation and proliferation in MGs.

Signaling Pathways	Experimental Model	Function
Hedgehog [16]	RMGECs	Proliferation and differentiation of acini
ADRB2/PKA [38]	HMGECs	Meibocyte differentiation and lipid synthesis
AKT [39]	HMGECs	Promote cell survival
STAT6/PPAR γ [40]	HMGECs	Meibocyte differentiation and lipid synthesis
SRC [41]	MOUSE	Meibocyte cell renewal and lipid synthesis
Shh [42]	MOUSE	Meibocyte differentiation and lipid synthesis

RMGECs: rat meibomian gland epithelial cells; HMGECs: human meibomian gland epithelial cells.

**Table 2:** Pathological mechanism of MGD.

Author [Year]	Pathological Mechanism	Comments
Liu et al. [2011] [61]	Genes regulated	MGD is associated with the overexpression of keratinization-related genes
Knop et al. [2011] [11]	Hyperkeratinization of the ductal system	MGD is mainly caused by the hyperkeratinization of the meibomian duct and orifice, accompanied with increased viscosity in the meibum
Parfitt et al. [2011] [62]	Aged-related acinar atrophy	MGD most likely results from glandular atrophy caused by the loss of meibocyte progenitors rather than ductal hyperkeratinization and gland obstruction
Jester et al. [2015] [9]	Gland atrophy	Consistent results indicate MGD without evidence of hyperkeratinization, implying that gland atrophy may be a major cause
Hwang et al. [2017] [10]	Meibocyte differentiation and renewal of acinar cells	PPAR-signaling-pathway-mediated abnormalities in meibocyte differentiation and renewal are the main cause of MGD

## Data Availability

This is a perspective, no new data was generated.
